# A Review on Treatment of Premature Ovarian Insufficiency: Characteristics, Limitations, and Challenges of Stem Cell versus ExosomeTherapy

**DOI:** 10.1155/2023/5760011

**Published:** 2023-11-17

**Authors:** Narges Elahi, Jafar Ai, Zohreh Makoolati

**Affiliations:** ^1^Department of Tissue Engineering, School of Advanced Technologies in Medicine, Fasa University of Medical Sciences, Fasa, Iran; ^2^Department of Tissue Engineering, School of Advanced Technologies in Medicine, Tehran University of Medical Sciences, Tehran, Iran; ^3^Department of Anatomical Sciences, Faculty of Medicine, Fasa University of Medical Sciences, Fasa, Iran

## Abstract

Premature ovarian insufficiency (POI) is a complex disorder that can result in varying degrees of infertility. Recently, mesenchymal stem cell (MSC) therapy and its derivatives, such as exosomes, have been introduced as novel strategies for the treatment of POI. This review discusses the features, limitations, and challenges of MSC and exosome therapy in the treatment of POI and provides readers with new insights for comparing and selecting chemical agents, optimizing doses, and other factors involved in study design and treatment strategies. MSC therapy has been shown to improve ovarian function in some animals with POI, but it can also have side effects such as high cost, time-consuming processes, limited lifespan and cell sources, loss of original characteristics during *in vitro* proliferation, dependence on specific culture environments, potential immune reactions, unknown therapeutic mechanisms, etc. However, exosome therapy is a newer therapy that has not been studied as extensively as MSC therapy, but that it has shown some promise in animal studies. The evidence for the effectiveness of MSC and exosome therapy is still limited, and more research is needed to determine whether these therapies are effective and safe for women with POI. This study presents a new perspective for researchers to advance their research in the fields of cell-based and cell-free therapies.

## 1. Introduction

Premature ovarian failure (POF) is a disorder in which women under 40 years of age experience 4–6 months of amenorrhea with high levels of FSH (follicle-stimulating hormone) and low levels of estradiol [1]. The global overall prevalence of POF is 3.5% among women. Furthermore, subgroup analysis shows a prevalence of 11.2% for iatrogenic etiology and 10.5% for autoimmunity [2]. The more precise term for this disorder is premature ovarian insufficiency (POI). At any stage of POI, a woman's fertility may be impacted by a drop in the number of early primordial follicles, an increase in follicle destruction or a decrease in the number of apoptotic follicles, and the inability of follicles to respond to gonadotrophin stimulation [1]. Various strategies have been employed to remedy POI. Hormone replacement therapy has been the first-line strategy, followed by more novel treatments such as stem cell and exosome therapy [3].

Stem cell therapy involves transplanting stem cells from sources like bone marrow, adipose tissue, or umbilical cord blood to promote ovarian tissue regeneration and restore function [4]. Reports suggest MSCs inhibit granulosa cell apoptosis and upregulate anti-Müllerian hormone and FSH receptor expression, offering hope for POF patients and infertile women [5]. Moreover, growth differentiation factor-9 (GDF-9) and stem cell factor (SCF) impact follicle development beyond the primary stage, with GDF-9 promoting primordial follicle formation and growth while FSH influences granulosa cell development via SCF in an animal model [6]. Exosome treatment, on the other hand, is a noncellular therapeutic strategy that takes advantage of the biologically potent characteristics of exosomes, which are nanosized extracellular vesicles released by cells. Proteins, nucleic acids, growth factors, and other bioactive substances that can affect the ovarian microenvironment and promote tissue regeneration are contained within exosomes' cargo [7]. The paracrine effects of exosomes allow for intercellular communication and targeted delivery of bioactive cargo, thus promoting tissue repair [8].

## 2. POI

POI is a heterogeneous disorder caused by genetic factors, autoimmune diseases, mitochondrial abnormalities, iatrogenic factors (including chemotherapy, radiotherapy, and surgical procedures), and environmental factors [3]. While over 50 genes are known to be related to POI, many cases still lack a clear genetic explanation [9]. Premature ovarian insufficiency can be treated using a variety of techniques. Although estrogen is thought to be physiologically replaced by hormone replacement treatment (HRT), ovarian function is not recovered. In vitro activation (IVA), mitochondrial activation, stem cell and exosome therapy, biomaterials techniques, and intraovarian platelet-rich plasma (PRP) injection are promising developing treatments for POI treatment. These innovative medicines are still in the experimental stage, despite their potential. A thorough assessment of their efficacy and safety is essential before they can be taken into consideration as viable clinical solutions [3]. In addition, identifying a marker like anti-Müllerian hormone (AMH) could aid in diagnosing and counseling women at risk for POI and assessing their ovarian reserve. AMH, produced by young ovarian follicles, is strongly correlated with their number, making it a potential diagnostic tool. Furthermore, AMH levels can indicate the degree of gonadal damage in cancer survivors [10].

## 3. Stem Cell Therapy

**Figure 1:**
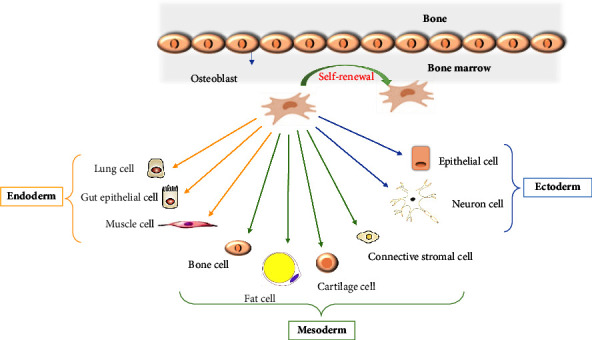
The multipotency properties of MSCs. This image illustrates differentiation and self-renewal (curved arrow). Ability of MSCs in the bone-marrow cavity *in vivo*.

Mesenchymal stem cell therapy, which is used in stem cell therapy, has lately attracted attention for restoring ovarian function in POI. A multipotent and diverse population of cells known as mesenchymal stem cells (MSCs) can develop along the mesodermal lineage (as shown in Figure 1). Hematopoietic markers (CD45, CD34, and CD14) and costimulatory molecules (CD80, CD86, and CD40) are not expressed by these cells. Human MSCs' expression of CD105, CD73, CD71, CD44, CD271, and CD90 is influenced by the tissue source and culture environment. According to evidence, MSCs release soluble substances such as transforming growth factor-1, interleukin-10 (IL-10), IL-6, and hepatocyte growth factor (HGF) on a regular basis. By inhibiting antigen-specific T-cell proliferation and encouraging the development of regulatory T cells, MSCs have an immunomodulatory effect. Successful allogeneic transplants have been thoroughly studied due to MSCs' low immunogenicity. When administered *in vivo*, MSCs have the capacity to stimulate peripheral tolerance. Furthermore, MSCs are intriguing agents for both local and systematic distribution since they may move from blood arteries to the target using their own unique surface molecules. The clinical therapeutic effectiveness of MSCs, however, mostly rests on their capacity to change the environment of wounded tissue through the physiological activity of stromal cells in the hematopoietic stem cell niche through the secretion of anti-inflammatory and antiapoptotic chemicals. Despite the widespread use of MSCs, there is debate concerning the unidentified long-term negative effects on immune function and tumorigenic potential [11]. The therapeutic benefits of MSC-based therapy are largely attributed to the effects of paracrine factors that promote angiogenesis. However, in aged MSCs, the secretion of these proangiogenic factors, including vascular endothelial growth factor (VEGF), placental growth factor (PGF), and HGF, is reduced. Conversely, the secretion of antiangiogenic factors such as thrombospondin-1 (TBS1) and plasminogen activator inhibitor-1 (PAI-1) is increased. As a result, aging has a detrimental impact on angiogenesis and directly undermines the therapeutic effectiveness of MSCs [12].

Human umbilical cord mesenchymal stem cells (UC-MSCs), which are obtained from the umbilical cord, possess the aforementioned features of MSCs, as well as a younger nature, lower tumorigenicity, and fewer ethical issues [13]. Umbilical cords are a great source for easily extracting mesenchymal stem cells. UC-MSCs express human leukocyte antigen major histocompatibility complex I (MHC I) at a low level, as well as CD29, CD73, CD105, and CD90. They do not express MHC II molecules, CD14, CD79, CD34, CD45, or HLA-DR, which gives these cells negligible immunogenic features [14, 15]. UC-MSCs possess immunomodulatory effects by influencing the differentiation, proliferation, and activation of T cell subsets while inhibiting B cell proliferation, differentiation, and other immune cell activities. They exhibit robust proliferative abilities and can differentiate into various cell types under suitable conditions, both *in vivo* and *in vitro*. In addition, UC-MSCs contribute to tissue repair and regeneration by secreting growth factors such as HGF, VEGF, stromal cell-derived factor-1 (SDF-1), keratinocyte growth factor (KGF), fibroblast growth factor (FGF), and insulin-like growth factor-1 (IGF-1), which help facilitate cell proliferation and tissue healing. UC-MSCs also play a role in mitigating inflammation at the site of injury, and they actively migrate to the injured site for repair. This migration is known as the “return” of the MSCs' “nest function” and has been demonstrated prominently in animal experiments under various microenvironmental conditions [16].

It has been demonstrated that UC-MSCs can improve the phosphatidylinositol-3-kinase (PI3K)/Akt signaling in POF-induced rodents via the nerve growth factor (NGF)/TrKA pathway. The PI3K/Akt signaling pathway regulates the follicular growth, survival, maturation, and differentiation of primordial follicles, as well as the prevention of apoptosis. Furthermore, the nerve growth factor receptor (TrkA) mainly activates the PI3K/Akt and mitogen-activated protein kinase (MAPK) signaling pathways, which are essential for the proliferation and survival of cells [15]. In a study, the intravenous injection of two doses of 1∗10^6^ UC-MSCs was able to transfer to the interstitium of the ovaries rather than to the follicles. This process prevented the apoptosis and inflammation in the granulosa cells in a POF-rodent model [13]. As shown in Table 1, different doses of UC-MSCs have been given to test their efficacy in restoring ovarian function in the POF model. UC-MSCs appear to have a large therapeutic potential. More consideration must be given to their therapeutic effectiveness, as well as to any potential drawbacks and negative side effects.

Female infertility brought on by degenerative factors is one condition for which bone marrow-derived mesenchymal stem cells (BMMSCs) have been touted as potential cures in regenerative medicine [22, 37]. Studies have shown that folliculogenesis may be affected by bone morphogenic proteins (BMPs), such as BMP-15 and BMP-6. These intraovarian subfamilies secreted from the oocyte have a key role in the development of follicles [19]. Vascular endothelial growth factor (VEGF), basic fibroblast growth factor (bFGF), insulin-like growth factor 1 (IGF-1), hepatocyte growth factor (HGF), and other cytokines and growth factors are among those secreted by BMMSCs. Angiogenesis, mitogenesis, and antiapoptotic events all depend on these variables. Granulosa cells (GCs) can create a new capillary network thanks to VEGF, an angiogenic cytokine factor. Folliculogenesis results from bFGF's stimulation of the promotion of primordial follicles. IGF-1 has the power to promote GC proliferation, inhibit apoptosis, and enhance antrum follicle development. HGF significantly affects follicle maturation and inhibits GC and follicle death in the ovary [22]. In this regard, BMMSCs have been applied for stem cell therapy in POF models.

During a woman's menstrual cycle, human endometrial mesenchymal stem cells (EnSCs) are a rich, noninvasive source of multipotent stem cells that can be used for autologous transplantation. In vitro, EnSCs develop quickly and can differentiate into a variety of cells depending on the particular cell environment. EnSCs express OCT4, CD9, CD29, CD105, SSEA-4, and CD73 but do not express CD34, CD133, HLA class I, or CD45 markers. It was demonstrated that the allogenic transplantation of ESCs is feasible for the dose-dependent regulation of mononuclear cell proliferation. Lai et al. applied EnSCs to investigate their therapeutic effect on ovary preservation in POI models. It was shown that EnSCs could recover the ovarian follicles after chemotherapy in animals [23].

Another potential treatment option for female reproductive issues caused by POI is MSCs generated from human embryonic stem cells (hESC-MSCs). In rats whose ovaries had been damaged by chemotherapy, it was shown that this kind of MSC may stop ovarian apoptosis and encourage ovulation. HESC-MSCs have been made available as an easy-to-expand cell type with a consistent population [19, 23, 38]. Nevertheless, despite all the research and benefits of hESC-MSCs, it is understandable to be concerned about their drawbacks and moral dilemmas. According to studies, hESC-MSCs have the capacity to develop germ-layer malignancies. The defective transplantation of stem cells derived from embryonic lines may lead to incomplete differentiation. Furthermore, genetic anomalies have been reported in hESC lines in long-term culture [38].

Fetal MSCs can be derived from the liver in early gestation and have long lifespans with appropriate immunomodulatory features. It was reported that FMSCs could restore ovarian function in POI-induced mice, as well as promote human granulosa cell proliferation through the melatonin membrane receptor 1 (MT1). The evidence suggests that the interaction of melatonin with its receptors (MT1 and MT2) leads to a reduction in the level of reactive oxygen species and the prevention of apoptosis, so that the MT1 and MT2 blockages affect the follicular atresia and porcine GCs and consequently the reproduction mediation. On the other hand, the antioxidant properties of melatonin and the MT1 receptor on cytoprotective activity have been reported in cisplatin-induced ovary injury [28].

The therapeutic effect of adipose-derived stem cells (ADSCs) on ovary failure due to cyclophosphamide (CTX) has been investigated. The results showed the effectiveness of these types of multipotent stem cells on ovulation and folliculogenesis [29, 30]. According to Cil et al., ADSCs have a specific impact on the phosphorylated-mTOR (p-mTOR) and mammalian target of rapamycin (mTOR), which are crucial for oocyte meiosis [30].

Furthermore, the chorionic plate-derived MSCs (CP-MSCs), the multipotent self-renewal adult stem cell, have been applied for the recovery of ovarian function through stimulating ovulation and folliculogenesis in POI models. These cells can be easily extracted from the chorionic plate of the human placenta, which is considered medical waste [31]. However, the therapeutic effect of autologous MSCs in women with POF disorder suggests a novel strategy that could decline the symptoms of menopause as well as estrogen enhancement. Despite the promising therapy, this study was limited to a few numbers of cases and requires long-term monitoring [39].

In recent years, clinical trials have been conducted on MSCs for various conditions, including autoimmune [40], neurodegenerative, cardiovascular, and bone and cartilage diseases. However, the number of approved MSC treatments worldwide remains limited. Interestingly, Asian countries have approved a higher number of MSC treatments compared to other countries [41].

Overall, when transplanted, MSCs possess the ability to migrate towards injured ovaries, promoting the restoration of secretory function, facilitating follicle formation, and promoting tissue reconstruction in POI models. Similar to white blood cells, MSCs express different receptors and cell adhesion molecules that assist in their migration towards the targeted organs, specifically injured ovaries. Crucially, specific chemokines bind to MSC receptors, guiding their movement towards the desired tissues. This migratory characteristic makes MSCs an excellent choice for regenerative therapies in POI. Once they migrate to the injured ovary, MSCs play a significant role in regulating ovarian cell proliferation, apoptosis, immune response, and oxidative stress through their paracrine effects. This highlights the critical importance of MSC migration as a key mechanism for enhancing the effectiveness of therapeutic interventions.

Table 1 provides a comprehensive overview of the molecular mechanisms underlying the effectiveness of MSC therapy for POI. Multiple studies emphasize the positive effects of various MSC types on ovarian function. Notably, MSCs have been found to reduce the secretion of inflammatory cytokines and FSH, which are often elevated in POI. Conversely, MSCs promote an increase in estrogen levels, anti-Müllerian hormone, and demonstrate improvements in the PI3k/AKT pathway, enhancing angiogenesis within the ovary. Moreover, MSCs extend their impact beyond hormone regulation. They play a critical role in inducing and supporting follicular growth, preventing follicular atresia, and inhibiting apoptosis. Together, these orchestrated biological effects hold immense potential for rejuvenating and restoring ovarian function in individuals with POI.

### 3.1. Stem Cell Therapy Limitations and Prospects

The therapeutic use of MSCs has faced a number of difficulties while receiving a lot of attention for the treatment of numerous illnesses, including those affecting the female reproductive system. The authors have identified some of these issues, as shown in Table 1. Controlling the quality of MSCs is difficult. While some groups' extraction procedures are time-consuming, intrusive, and expensive, others may lose their original characteristics while proliferating in vitro. Other challenges include a short lifespan and cell sources, unidentified therapeutic methods, the tumorigenicity of stem cell therapy, unclear dosing frequency, and particular growth conditions. Especially in the case of a disease state, it is urgent to have an appropriate and precise estimation for a sufficient number of cells in the transplantation process, which may be affected by apoptosis, inflammation, and any special condition of the POI disorder. Over the years, various amounts of stem cells have been administered for POI treatment, and the exact amount has not been determined. However, it seems essential to define the stage of POI first based on the chemotherapy agents and their doses, taking into account any probable adverse effects in the long term. Then, try to use standard concentrations, protocols, and materials for administration. Furthermore, evidence has shown that the physiochemical and mechanical features of the surrounding microenvironment of primordial germ line cells have a significant effect on their fate, growth, maturation, and differentiation. In fact, a three-dimensional structure and biomechanical properties are provided by the natural extracellular matrix (ECM), which has a great role in signaling phenomena, cell-to-cell communication, and consequently tissue development [42, 43]. In this line, alginate-ECM gels have been used to illustrate the role of ECM and its components in regulating the development of follicles [43].

In addition, scaffold-based stem cell transport has been developed to circumvent the main drawbacks of stem cell therapy. The extremely low cell survival rate in cell treatment is seen as a serious issue. The survival, adhesion, proliferation, and differentiation of stem cells must therefore be enhanced by providing a milieu that is similar to the cell niche. Collagen and alginate are examples of natural-based scaffolds that have recently been developed and demonstrated to have the ability to awaken POI follicles that are in a dormant state [33, 35, 36, 44].

## 4. Exosome Therapy

Exosomes, which are the nanosized extracellular vesicles produced within eukaryotic cell endosomes, have gained significant attention in the fields of life sciences research and biotechnology [45]. They play a significant role in cell-to-cell communication, signaling, and consequently physiological cellular action and development [46]. Exosomes are generated through the fusion of exosome-containing endosomes with the plasma membrane, whereas the secretion of microvesicles and apoptotic bodies occurs through direct budding from the plasma membrane. The biogenesis of exosomes starts with inward budding of the plasma membrane, forming early endosomes, and progresses to the maturation of multivesicular bodies (MVBs), wherein intraluminal vesicles (ILVs) are formed by inward budding of the endosomal membrane. These ILVs contain lipids, proteins, and nucleic acids derived from their parent cells [45]. The heterogeneous vesicles are categorized into exosomes, apoptotic bodies, and microvesicles based on their size and biogenesis [46]. When processing and separating exosomes, it is crucial to take into account variables including the makeup of the initial sample, the chosen method of separation, and how these variables affect the quality and traits of the finished products. Ultracentrifugation, ultrafiltration, precipitation, immunoaffinity capture, and size-exclusion chromatography are the five methods that are frequently employed for exosome processing. These techniques all generate exosomes, however, to varied degrees of purity and number. Combining isolation methods is a typical strategy to increase exosome yield and purity [45]. Exosomes are secreted in physiological and pathological states and are present in follicular fluid (FF). It has been reported that hormonal response, oocyte differentiation, follicular growth, and the meiosis onset pathways have been regulated by the involvement of FF exosomes [46]. Furthermore, the evidence has demonstrated that exosomes carry a variety of microRNAs (miRNAs), some of which, such as miR-100, miR-132, miR-212, and miR-214, directly regulate the meiosis and maturation of follicles [47].

MiRNAs are small noncoding regulatory RNAs that function in posttranscriptional gene regulation with the ability to regulate cellular processes broadly [48]. Recent studies have shown that MSC-derived exosomes can promote tissue repair and regeneration, making them an attractive candidate for the treatment of POI (Table 2). Studies have shown that the therapeutic effects of MSCs may be due to their paracrine factors, which include exosomes. These bilayered structures appear to have the capacity to overcome some limitations of MSCs, such as vascular blockage due to the large size of cells or finite lifespan and sources. However, it has been noted that a newly introduced method may overcome the challenge of low extracted numbers of exosomes. Cha et al. reported that their 3D-bioprocessing method has the potential to produce efficiently scalable EVs from human MSCs for clinical and/or commercial applications [63].

**Figure 2:**
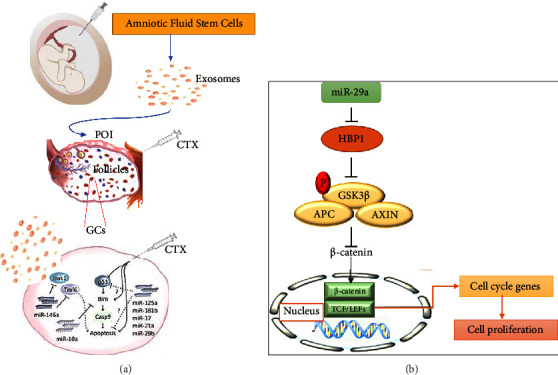
The suggested mechanisms of MSCs-derived exosomes in the recovery of ovarian function in POF model. (a) The mechanism of AFMSCs-derived exosomes contain various significant miRNA-10a on damaged GCs. (b) HUCMSCs exosomal miRNA-29a effect on cell proliferation through the Wnt/*β*-catenin pathway.

**Figure 3:**
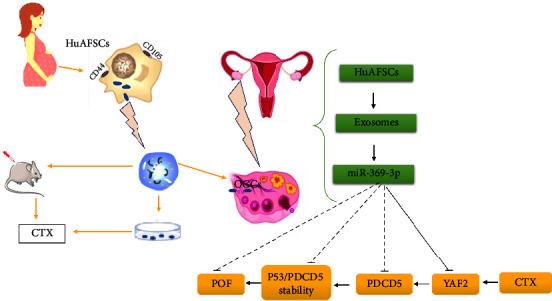
CD44^+^/CD105^+^HAFMSCs-exosomes carrying miR-369-3p have the capacity to specifically downregulate the expression of YAF2, prevent the stability of PDCD5/p53, and decrease the apoptosis of GCs, leading to the restorative effect on POF.

In addition, research on FF EVs has shown that distinct miRNA types depend on the size and type of follicles. The types of miRNAs shift from those associated with cell proliferation pathways to those associated with inflammatory response pathways when follicles develop and differentiate into bigger antral ones [64]. Small extracellular vesicles derived from embryonic stem cells have the potential to promote the recovery of ovaries in POF mice models by improving folliculogenesis and the proliferation of GCs through the PI3K/AKT signaling pathway [49]. MiRNA-21, one of the many miRNAs, has been shown to play a significant role in ovarian folliculogenesis by controlling and interacting with a variety of target genes. Consequently, autoimmune POIs have low levels of miRNA-21 expression. MiR-21 has demonstrated a positive link with AMH, E2, uterine size, and ovarian volume in a POI mice model and a negative correlation with FSH and LH [65]. Thabet et al. studied amniotic fluid mesenchymal stem cells (AFMSCs) to determine the exosomal miRNA. They found that AFMSCs-derived vesicles are a great source of miRNA-21, which prevents apoptosis, induces follicle regeneration, and recovers ovary function in infertile rats through the phosphatase and tensin homolog (PTEN) and PI3K/PTEN pathways [48]. Furthermore, AFMSCs-derived exosomes contain miRNA-146a and significantly miRNA-10a, which have antiapoptotic effects and inhibit ovarian follicles from atresia in CPA-induced animal models [50].  Figure 2(a) shows the schematic diagram of the proposed mechanism of AFMSCs-derived miRNA-146a on damaged GCs. The exosomal miRNA-369-3p from AFMSCs has similar behavior in POF models through a specific pathway (Figure 3) [52]. Another study showed that human amniotic epithelial cell-derived exosomes include a variety of miRNAs, such as miRNA-1246. HAEC-exosomes have been administered to investigate their effect on ovarian follicles against apoptosis and have shown significant efficacy on folliculogenesis [66]. It has been reported that there is an interaction between miRNA-17-5P and sirtuin-7 (SIRT7), which can be extracted from HUCMSCs in the ovary. SIRT7 regulates the response of cells to metabolic, oxidative, and genotoxic stresses. In a POI model, the administration of miRNA-17-5P could restore ovarian function, trigger GC proliferation, reduce ROS accumulation, and inhibit SIRT7 expression [53].

HUCMSCs exosomal miRNA-100-5p has acted through the NOX4/NLRP3 signaling pathway to prevent apoptotic phenomena in a POF model [67]. HUCMSCs exosomal miRNA-29a (Figure 2(b)) [54] and HUCMSCs-derived exosomes [56, 68] have promoted ovarian function, improved angiogenesis, developed folliculogenesis, and restored the estrous cycle through the Wnt/*β*-catenin, Hippo, and PI3K/AKT signaling pathways in POI-induced rodents, respectively. In addition, exosomal miRNA-29a derived from HUCMSCs promotes GC growth and angiogenesis in cisplatin-induced rats [55].

HUCMSCs exosomal miRNA-126-3p prevents GC apoptosis through the PIK3R2/PI3K/AKT/mTOR axis *in vitro* [55]. Exosomes derived from HADSCs have the potential to target the SMAD/TGF*β* signaling pathway, leading to the proliferation of oocytes and GCs, promoting hormonal secretion and follicle differentiation [58]. The secretome of human bone marrow MSCs has also been investigated for POF treatment. The findings show that this secretome contains exosomal miR-144-5p [59] and miR-644-5p [60], which target PTEN and p53 to prevent GC apoptosis, respectively. Furthermore, the human BMMSC secretome significantly affects GCs, thus improving the secretion of GC hormones and stimulating growth and proliferation [69].

Menstrual blood-derived stromal cells (MenSCs), according to studies, can help with fertility restoration. In this regard, a rat model was used to study the therapeutic effects of exosomes produced from MenSCs. MenSCs-exosomes could enhance ovarian function, restore the ovary cortex, and encourage GC proliferation, according to in vivo research [61].

However, there still exists a lack of clarity concerning the complete substitution of MSCs by exosomes in the treatment of POI. Establishing the reliable implementation of cell-free therapy utilizing exosomes for POI patients necessitates investigating potential disparities in outcomes and effectiveness between MSC and MSC-derived exosome treatments. In a conducted study, samples of tissue and serum were acquired subsequent to MSC/exosome therapy to evaluate molecular changes resulting from the treatment. Furthermore, parallel breeding experiments were conducted to compare the restoration of fertility. Both the MSC-treated and exosome-treated groups exhibited reestablished estrous cycles and serum hormone levels compared to untreated mice with POI. Following treatment, the pregnancy rate in the MSC-treated group ranged from 60 to 100%, while the exosome-treated group demonstrated a pregnancy rate of 30 to 50%. Interestingly, regarding long-term effects, the MSC-treated mice consistently maintained a pregnancy rate of 60 to 80% during a second breeding cycle, whereas the exosome-treated group experienced a recurrence of infertility during the second breeding round [70].

In summary, studies have demonstrated the obvious benefits of exosome therapeutics in regenerative medicine and for the treatment of premature ovarian insufficiency. However, preclinical trials demonstrating the efficacy and safety of exosome therapy for POI are lacking.

### 4.1. Exosome Therapy Limitations and Prospects

Although cell-free techniques like exosome therapy are receiving more attention, there are still significant obstacles to their usage in POI treatment. Exosomes are excellent suppliers of a variety of miRNAs and other compounds that might modify cellular function, as was previously described. It will need additional research to ascertain the precise mechanism of each miRNA as well as any potential connections between exosomes and other signaling pathways. Determining the precise processes, how they work together, and most crucially, how they relate to the molecules involved in inflammation, regulation, and immunomodulation is also crucial. As discussed regarding the challenges of stem cell therapy in POI treatment, there is a significant gap in the design of models, applied materials and drugs, and treatment strategies, as well as exosome therapy. The other main challenges are the lack of standard protocols for the isolation, purification, and characterization of MSC-derived exosomes, which can lead to variability in the quality and potency of exosomes used for therapy. Exosome-based therapies' safety must also be carefully considered because there is a chance of immune rejection and unintended side effects. In addition, there is no agreement on the administration strategy. Exosomes that are administered conventionally have been demonstrated to quickly leave the bloodstream; 2 hours after injection, they were found in the liver, spleen, lung, and gastrointestinal systems. For the optimum performance of local delivery of exosomes, Riau et al. suggested sustained distribution of exosomes using biodegradable materials like hydrogel [71]. Finally, there is no research that determines any probable long-term and systematic administration effects of miRNAs on reproductive diseases. Thus, it is essential to consider the effect of these vesicles on other organs due to their nanosize (Table 3).

## 5. Conclusion

In conclusion, mesenchymal stem cells and MSC-derived exosomes hold great promise as potential therapeutic options for the treatment of premature ovarian insufficiency. In combination with the wide variety of miRNAs and other chemicals found in exosomes, MSCs have the unusual capacity to specialize into a variety of cell types, which makes them excellent candidates for regaining ovarian function. To effectively utilize their therapeutic potential, a number of issues and restrictions must be resolved. The absence of established techniques for the isolation, growth, safety, and characterization of MSCs and exosomes is one of the major difficulties. The viability, efficacy, and reproducibility of the treatments may be impacted by this variation in methodology. Controlling MSC quality is also important because in vitro proliferation and extraction methods can change their original characteristics. In order to guarantee enough cell counts for transplantation, specified parameters and administration concentrations must be established while taking into account variables like the stage of POI and potential long-term negative consequences. Furthermore, the fate, development, and differentiation of primordial germ line cells are significantly influenced by the physiochemical and mechanical characteristics of the milieu around them. Collagen and alginate are examples of natural-based scaffolds that have shown potential for improving the viability and stimulation of dormant follicles in POI. Scaffold-based delivery systems offer a milieu that resembles the cell niche, enhancing stem cells' adhesion, proliferation, and differentiation. Exosome therapy has drawn interest as a cell-free approach; however, there are still issues to be solved. Exosomal miRNAs' precise mechanisms of action and interactions with other signaling pathways must be clarified through further study, which is now underway. This information will be useful in developing more potent therapy strategies for POI. Taking everything into account, it is clear that while MSCs and exosomes produced from MSCs have a great deal of potential to treat POI, further study is required to overcome current obstacles and improve the delivery, characterization, and administration protocols. Taking on these issues will open the door for the creation of efficient, standardized, and secure therapy approaches for women with POI.

## Figures and Tables

**Table 1:** Various types of MSCs application in POI treatment.

Type of MSCs	Route of administration	Amount of administration	Administered drug to induce POF	Effect	Limitation	Animal model	Ref
Human umbilical cord mesenchymal stem cell (HUC-MSCs)	Tail veins intravenously (IV), twice	1 × 10^6^	120 mg/kg of CTX and 30 mg/kg busulfan	Antiapoptotic and anti-inflammatory, inhibition of GC apoptosis and inflammation	Unknown administration frequency, undetermined fate of UC-MSCs *in vivo*, and undetermined pharmacokinetics of UC-MSCs *in vivo*	Mice	[13]
HUC-MSCs	Tail veins (IV)	1 × 10^6^	Cisplatin for 7 days, 2 mg/kg of cisplatin (intraperitoneally)	Enhance follicular development and restore the ovarian function, inhibit theca interstitial cells apoptosis by regulating NR4A1-mediated mitochondrial mechanisms		Rat	[17]
HUCMSCs	Tail veins (IV)	2 × 10^6^	30 mg/kg busulfan and 120 mg/kg CTX	Recovered disturbed hormone secretion, FSH, and AMH	Need further investigations to confirm the mechanism involved in the ovarian function recovery	Mice	[18]
HUCMSCs	IV and *in situ*	25 *μ*L, at a concentration of 2 × 10^6^/mL UC-MSCs in situ 1 × 10^6^/mL intravenously with a microinjector	200 mg/kg of CTX on the first day and then 8 mg/kg/day for the 15 consecutive days	Recovered disturbed hormone secretion and folliculogenesis	Restoration of the ovarian function takes place to some degree, tumorigenic potential of stem cells therapy, follow the ultimate fate of these cells, investigate the fertility of the rat model following our current CTX administration protocol and UC-MSCs therapy and require better understanding of the exact mechanism	Rat	[14]
HUCMSCs	Tail vein (IV)	5 × 10^6^	50 mg/kg CTX on the first experiment day, followed by 8 mg/kg/day for 14 days	Partially recovered disturbed hormone secretion and folliculogenesis via the NGF/TrkA signaling pathway	Tumorigenicity of these cells, requires deeper investigations to better understand the exact underlying mechanism and the safety of the therapeutic effects of UC-MSCs on POF	Rat	[15]
Ovarian stromal stem cells (OSSCs)	Intraperitoneally the second and ninth days of the study	4 × 10^6^	A dose of 200 mg/kg of CTX	Follicle maturation	The source of OSSCs, no standardization different for chemical agents and their concentrations, investigate the cytotoxicity of applied agents, generate the effective and standardized method	Rat	[19]
Bone marrow derived mesenchymal (BMMSCs)	Intraperitoneally the second and ninth days of the study	4 × 10^6^	A dose of 200 mg/kg of CTX	Follicle maturation	The source of OSSCs, no standardization different for chemical agents and their concentrations, investigate the cytotoxicity of applied agents, generate the effective and standardized method	Rat	[19]
BMMSCs	On day 4 after CTX injection through the tail vein	0.5 × 10^6^	A dose of 80 mg/kg of CTX	A drop in estradiol and rise in follicle-stimulating hormone and E2 levels	Should be repeated in a more tightly controlled way	Mice	[20]
BMMSCs	The day after CTX injection (days 9 and 16) intraperitoneally	4 × 10^6^/kg	CTX 200 mg/kg intraperitoneally on the eighth and fifteenth days of the study	Be protective from germ cell apoptosis and DNA damage	The transplanted rats observed for only two weeks. Need to follow for long-term effects of the treatment	Rat	[21]
BMMSCs	Directly injected into the bilateral ovaries	2 × 10^6^	50 mg/kg of CTX on the first day, then 8 mg/kg/day for 13 consecutive days (a total of 14 doses)	Be protective from germ cell apoptosis and DNA damage, a drop in estradiol and rise in follicle-stimulating hormone and E2 levels	BMSCs-secretome is likely responsible for the therapeutic paracrine effect of BMSCs. Stem cell secretome is expected to overcome the limitations of stem cell transplantation and become the basis of a novel therapy for ovarian damage	Rat	[22]
Human endometrial mesenchymal stem cells (EnSCs)	Tail vein	2 × 10^6^	Intraperitoneal injection of busulfan (30 mg/kg; and CTX 120 mg/kg)	Reducing the depletion of the germline stem cell (GSCs) pool induced by chemotherapy		Mice	[23]
Human menstrual blood-derived stromal cells (MenSCs)	Through tail vein injection in seven days after CTX treatment	1 × 10^6^	Intraperitoneal injection of 70 mg/kg CTX at the age of seven weeks	Regulating normal follicle development and estrous cycle, reducing apoptosis in ovaries to maintain homeostasis of microenvironment and modulating serum sex hormones to a relatively normal status. Participated in the activation of ovarian transcriptional expression in the ECM-dependent FAK/AKT signaling pathway and thus restoring the ovarian function to a certain extent		Mice	[24]
Human embryonic stem cell-derived mesenchymal stem cells (hES-MSCs)	Tail vein	5 × 10^6^	2 mg/kg cisplatin daily for 10 days	Folliculogenesis be protective from germ cell apoptosis		Mice	[25]
HES-MSCs	IV	1 × 10^6^	100 mg/kg CTX for 10 consecutive days	Restored the injured ovary by cytokine suppression of granulosa cell apoptosis and increased the follicular growth		Mice	[26]
HAMSCS	Intraperitoneally	1 × 10^6^	The bilateral ovaries were burned for 30 s∼1 min with 10% hydrogen peroxide	Recovery of the estrus cycle, estrogen levels increased, while follicle-stimulating hormone levels decreased. Increasing of the ovarian index, fertility rate, and population of follicles at different stages. No obvious deformity in newborn mice and showing normal growth and development		Mice	[27]
Fetal liver mesenchymal stem cells	IV	1 × 10^6^ FMSCs 2 weeks after CTX injection	A single intraperitoneal injection of CTX, 120 mg/kg injected everyday continued for 2 weeks	Preventing CTX-induced follicle loss and recovering sex hormone levels. Decreasing oxidative damage, increasing oxidative protection, improving antiapoptotic effects, and inhibiting apoptotic genes. Stimulating the activity of POI hGCs by targeting MT1		Mice	[28]
Adipose-derived stem cells	IV and *in situ*	1 × 10^6^ (IV), 1 × 10^5^ (injected directly into the bilateral ovaries)	50 mg/kg CTX for 15 consecutive days of injection	Increasing the population of follicles at different stages and ovulation		Mice	[29]
Adipose-derived mesenchymal stem	Injected locally into the ovary	5 × 10^4^	50 mg/kg CTX on the first day and at 8 mg/kg during the following 13 day	Inhibiting the loss of mTOR and p-mTOR signaling, which is key to meiosis in oocytes		Rat	[30]
Human chorionic plate-derived mesenchymal stem cells	Intravenously transplanted into the mice once a week for 4 weeks	2 × 10^6^ cells/kg	50 mg/kg CTX for 15 consecutive days	A drop in estradiol and rise in follicle-stimulating hormone and E2 levels and folliculogenesis		Mice	[31]
Clonal mesenchymal stromal cells	Intravenously transplanted into the mice	100 *μ*l of PBS containing 1 × 10^6^ cells	50 mg/kg CTX for 15 consecutive days	Protection of granulosa cells from CTX-induced damage, improvement in the angiogenesis via upregulation expression of VEGF and IGF1 at the mRNA level and VEGF and *α*SMA at the protein level, inhibition of apoptosis through the PI3K/AKT signaling pathway	The effective dose requires further study for clinical trials	Mice	[32]
Human ESC-MPCs with PLGA/hyaluronic acid (HA) sponge	Intravenous injection or local administration	5 × 10^6^ cells/50 *μ*L PBS and 50 *μ*L HA gel	Cisplatin (2.0 mg/kg) for 10 days	Prolonging the cell survival rate *in vivo*. Recovered ovarian functions, including a significantly increased number of ovarian reserves, estrogen levels, and AMH levels and decreasing apoptotic levels. Improving the quality of oocytes and embryos and estrous cycle regularity		Mice	[33]
Human umbilical cord mesenchymal stem cell	Patients	0.5 × 10^7^/100 *μ*L, at three points, with 35 *μ*L of UC-MSCs per point		Increased follicular development and improved egg collection	More investigation to confirm the duration of stem cells efficacy, distinguishing more appropriated clinical cases fit for this therapy, validating the dose of UC-MSCs	Human	[34]
Umbilical cord–derived mesenchymal stem cells on a collagen scaffold	Suspensions (10 *μ*l) were injected into the core of the ovaries	2 × 105 UC-MSC in 10 *μ*l degradable collagen	CTX (40 mg/kg/day) for 15 consecutive days	Promoting ovarian angiogenesis with the increased expression of CD31	Unknown mechanism of interaction between collagen scaffolds and stem cells remains. Choose a proper density of stem cells on a collagen scaffold to allow cell to distribute evenly. Requires further investigation of the potential underlying mechanism of collagen scaffolds in UC-MSCs growth after transplantation	Mice	[35]
Umbilical cord mesenchymal stem cells on a collagen scaffold (collagen/UC-MSCs)	Patients	5 × 10^6^/400 *μ*L for unilateral ovarian injection, collagen concentration, 5 mg·mL^−1^		Rescuing overall ovarian function, elevating estradiol concentrations, improving follicular development, and increasing number of antral follicles. Successful clinical pregnancy in women with POF after transplantation of collagen/UC-MSCs or UC-MSCs		Human	[36]

**Table 2:** Various types of MSCs-derived exosomes application in POI treatment.

Type of MSCs	Route of administration	Amount of administration	Administered drug to induce POF	Effect	Limitation	Animal model	Ref
Embryonic stem cells-derived extracellular vesicles	The tail vein intravenously three times once every 2 days	1 × 10^8^/mL	CTX 120 mg/kg and busulfan 30 mg/kg	Recovery of the levels of serum sex hormones to normal levels, increasement of follicles, reduction of the number of apoptotic cells, and improvement of ovarian function by regulating the PI3K/AKT signaling pathway		Mice	[49]
AFMSCs- exosomes carrying miRNA-21	Intraovarian injection	0.5 × 10^6^	CTX (40 mg/kg)	Prevent the apoptosis phenomenon, induce the follicles regeneration and recover the ovary function in infertile rat through the phosphatase and tensin homolog (PTEN) and PI3K/PTEN pathway		Rat	[48]
AFMSCs- exosomes carrying miR10a	After CTX for 24 h, intra-ovarian injections	125 *μ*g	Busulfan 20 mg/kg and CTX 200 mg/kg	Anti-apoptotic effect on CTX-damaged GCs		Mice	[50]
Amniotic fluid-derived exosomes	Intraovarian injection	10 *μ*l	200 mg/kg CTX on the first day and then with 8 mg/kg/d consecutively for 14 days	Recover of the levels of serum sex hormones to normal levels; restoring the ovarian function through the TGF-*β*/Smads signaling pathway		Rat	[51]
CD44^+^/CD105^+^HAFSC-exosomes carrying miR-369-3p	Via the tail vein every 2 days for 4 weeks	1 × 10^6^ exosomes	CTX 70 mg/kg for 1 week, and then, CTX injection intraperitoneally at a dose of 30 mg/kg for 2 weeks every 2 days	Downregulate the expression of YAF2, inhibit the stability of PDCD5/p53, and reduce the apoptosis of ovarian granulosa cells	Role of miR-369-3p in AFSCs is limited, exploration for multiplicity of mechanisms, low yield of exosome, production efficiency must be increased	Mice	[52]
HUCMSCs- exosomes carrying miRNA-17-5p	Intraovarian injections	10^11^ particles/mL	A dose of 120 mg/kg CTX	Promoting proliferation of CTX-damaged GCs and ovarian cells, and alleviating ROS accumulation by delivering exosomal miR-17-5P and inhibiting SIRT7 expression		Mice	[53]
HUCMSCs- exosomes carrying MicroRNA-29a	Tail vein	Exosomes (125 *μ*g dissolved in 100 *μ*l PBS)	5 mg/kg cisplatin	Maturation of follicles, inhibition in GC apoptosis and activating the Wnt/*β*-catenin pathway	Limitation in number of animals	Mice	[54]
HUCMSCs- exosomes carrying miR-126-3p	A single dose via caudal vein after 14 days of injection of cisplatin	Of 400 *μ*g exosomal proteins/200 *μ*l PBS	Cisplatin (1 mg/kg) for 14 days	Promoting proliferation and inhibiting the apoptosis of OGCs PIK3R2/PI3K/AKT/mTOR axis in vitro, increasing E2 and AMH levels, increasing body and reproductive organ weights and follicle counts, and reduced FSH levels	Develop the exosome extraction strategies order to produce purer and higher volumes of membrane vesicles, use further test besides the morphologic and functional tests to evaluate ovary damage, evaluate the reproductive function in chemotherapy-induced POF models, need more experiments for any possible downstream molecules associated with miR-126-3p, follow the long-term effects of miR-126-3p-hucMSCs-exosomes and further evaluation of its efficacy and safety	Rat	[55]
HUCMSCs- -derived microvesicles	Injected into the vena caudalis	150 *μ*g	Busulfan (20 mg/kg) and CTX (200 mg/kg)	Promoting the ovarian angiogenesis, and recovering the disturbed estrous cycle, improving the numbers of primordial, developing, and preovulatory follicles	Requiring the natural mating trial would provide supplementary evidence to prove the effect of MVs in restoring damaged ovarian, exploring the molecular mechanisms involved in the angiogenesis promoting effects	Mice	[56]
HUCMSC-exosomes	Tail intravenous injection	1 ml PBS containing 1 × 10^10^ particles	CTX combined with busulfan	Alleviating ovarian injury, facilitating ovarian function restoration, and protecting fertility; improving the local microenvironment of ovarian tissue in POI rats through immune regulation, cellular viability, inflammation regulation, fibrosis, and metabolism			[57]
HADSC-exosomes		1 × 10^6^	CTX, 120 mg/kg, for 2 weeks	Proliferation of oocytes and GCs, promoting the hormonal secretion and follicles differentiation by SMAD/TGF*β* signaling		Mice	[58]
BMSC-derived exosome	Injected intraperitoneally every other day for 2 weeks	150 *μ*g	50 mg/kg CTX on the first day and then with 8 mg/kg/d consecutively for 14 days	Recovering the estrus cycle, increasing the number of basal and sinus follicles in POF rats, increasing estradiol (E2) and anti-Mullerian hormone (AMH) levels, and reducing follicle stimulating hormone (FSH) and luteinizing hormone (LH) levels in the serum, preventing the ovarian follicular atresia via the delivery of miR-144-5p, by targeting PTEN		Rat	[59]
BMSC-derived exosome	Injected into the tail vein on the 1st, 5th, and 10th day after modeling	125 *μ*g/100 *μ*L PBS	5 mg/kg cisplatin	Target regulation of p53 to inhibit ovarian granulosa cell apoptosis		Mice	[60]
MenSCs-exosomes	Intraovary injection	25 *μ*g	VCD (4-vinylcyclohexene diepoxide) for 15 consecutive days (80 mg/kg per day)	Promoting the ovarian reserve, serum hormones, and fertility	Further explorations require for the MenSCs-exosomes effects on oocyte-granulosa cross-talking or gap-junction, investigate the molecular mechanism, identify the active components of MenSCs-exosomes for improving ovarian function (such as protein or micro-RNA)	Rat	[61]
Clonal MSCs-derived extracellular vesicles	Intravenously transplanted into the mice	100 *μ*l of PBS containing 400 *μ*g EV	50 mg/kg CTX for 15 consecutive days	Protection of granulosa cells from CTX-induced damage, improvement of the angiogenesis via upregulation expression of VEGF and Igf1 at the mRNA level and VEGF and *α*SMA at the protein level, inhibition of apoptosis through the PI3K/AKT signaling pathway. EV20K is more cost-effective and feasible than EV110K	The full cargo and function of the isolated EVs are not yet well known. The effective dose requires further study for clinical trials	Mice	[32]
MenSCs-exosomes	Intraovary injection	50 *μ*l conditional exosomes suspended in PBS with about 4.5 × 10^8^ particles/ml	IP injection of 4-vinylcyclohexene diepoxide for 15 continuous days	Ameliorating granulosa cell apoptosis by regulating the SMAD3/AKT/MDM2/P53 pathway via delivery of thrombospondin-1		Rat	[62]

**Table 3:** Overall comparison description of the stem cell- versus exosome therapy in POI.

Effects, limitations & challenges	Type of treatment		
	Mesenchymal stem cell (MSC)	MSCs-derived exosome	Ref
Antiapoptotic and anti-inflammatory effects	^*∗*^	^*∗*^	[28, 33, 52]
Recovery of the secretion of the disturbed hormones	^*∗*^	^*∗*^	[14, 15, 33]
Recovery of folliculogenesis	^*∗*^	^*∗*^	[14, 15, 66]
Follicle maturation	^*∗*^		[19, 22]
Decreasing oxidative damage, increasing oxidative protection	^*∗*^	^*∗*^	[28, 53]
Requiring to prolong the cell survival rate *in vivo*	^*∗*^		[33]
Promoting the ovarian angiogenesis	^*∗*^	^*∗*^	[12, 35, 55]
Requiring the adequate cell sourcing	^*∗*^		[42, 43]
Safety concern	^*∗*^	^*∗*^	[3, 15, 55]
Follow up the long-term effect of transplanted cells/cargo	^*∗*^	^*∗*^	[55, 70]
Requiring to standard isolation and characterization methods		^*∗*^	[45]
Requiring to standard the administration method	^*∗*^	^*∗*^	[14, 71]
Unknown administration frequency	^*∗*^	^*∗*^	[13, 71]
Undetermined fate	^*∗*^	^*∗*^	[13, 14, 42, 43]
Undetermined pharmacokinetics *in vivo*	^*∗*^	^*∗*^	[13]
Tumorigenic potential	^*∗*^	?	[11, 13–15]
Requiring to understand the exact mechanism	^*∗*^	^*∗*^	[17, 18, 44, 56, 61]
The effective dose requires further study for clinical trials	^*∗*^	^*∗*^	[32, 38, 40]
Increasing the yield		^*∗*^	[52]
Needing to increase the production efficiency		^*∗*^	[52]

The asterisk (^∗^) indicates confirmation for each statement, while the question mark (?) indicates that there is no definitive answer.

## Data Availability

The data used to support the findings of this study are available from the corresponding author upon request.

## References

[1] Welt C. K. (2008). Primary ovarian insufficiency: a more accurate term for premature ovarian failure. *Clinical Endocrinology*.

[2] Li D. L., Zhu Y., Wei J., Chen L., Chen S. (2023). The global prevalence of premature ovarian insufficiency: a systematic review and meta-analysis. *Climacteric*.

[3] yi Huang Q., rong Chen S., ming Chen J., yang Shi Q., Lin S. (2022). Therapeutic options for premature ovarian insufficiency: an updated review. *Reproductive Biology and Endocrinology*.

[4] Fazeli Z., Abedindo A., Omrani M. D., Ghaderian S. M. H. (2018). Mesenchymal stem cells (MSCs) therapy for recovery of fertility: a systematic review. *Stem Cell Reviews and Reports*.

[5] Yoon S. Y. (2019). Mesenchymal stem cells for restoration of ovarian function. *Clinical and Experimental Reproductive Medicine*.

[6] Wang J., Roy S. K. (2004). Growth differentiation factor-9 and stem cell factor promote primordial follicle formation in the hamster: modulation by follicle-stimulating hormone. *Biology of Reproduction*.

[7] Yeo R. W. Y., Lai R. C., Zhang B. (2013). Mesenchymal stem cell: an efficient mass producer of exosomes for drug delivery. *Advanced Drug Delivery Reviews*.

[8] Baranyai T., Herczeg K., Onódi Z. (2015). Isolation of exosomes from blood plasma: qualitative and quantitative comparison of ultracentrifugation and size exclusion chromatography methods. *PLoS One*.

[9] Ishizuka B. (2021). Current understanding of the etiology, symptomatology, and treatment options in premature ovarian insufficiency (POI). *Frontiers in Endocrinology*.

[10] Visser J. A., Schipper I., Laven J. S. E., Themmen A. P. N. (2012). Anti-Müllerian hormone: an ovarian reserve marker in primary ovarian insufficiency. *Nature Reviews Endocrinology*.

[11] Uccelli A., Moretta L., Pistoia V. (2008). Mesenchymal stem cells in health and disease. *Nature Reviews Immunology*.

[12] Liu J., Ding Y., Liu Z., Liang X. (2020). Senescence in mesenchymal stem cells: functional alterations, molecular mechanisms, and rejuvenation strategies. *Frontiers in Cell and Developmental Biology*.

[13] Deng T., He J., Yao Q. (2021). Human umbilical cord mesenchymal stem cells improve ovarian function in chemotherapy-induced premature ovarian failure mice through inhibiting apoptosis and inflammation via a paracrine mechanism. *Reproductive Sciences*.

[14] Li H., Song D., Zhong Y. (2016). Human umbilical cord mesenchymal stem cells therapy in cyclophosphamide-induced premature ovarian failure rat model. *BioMed Research International*.

[15] Zheng Q., Fu X., Jiang J. (2019). Umbilical cord mesenchymal stem cell transplantation prevents chemotherapy-induced ovarian failure via the NGF/TrkA pathway in rats. *BioMed Research International*.

[16] Lv X., Wang L., Zou X., Huang S. (2021). Umbilical cord mesenchymal stem cell therapy for regenerative treatment of rheumatoid arthritis: opportunities and challenges. *Drug Design, Development and Therapy*.

[17] Luo H., Tang Y., Jiang Z., Bao H., Fu Q., Zhang (2022). hUCMSCs reduce theca interstitial cells apoptosis and restore ovarian function in premature ovarian insufficiency rats through regulating NR4A1-mediated mitochondrial mechanisms. *Reproductive Biology and Endocrinology*.

[18] Lv X., Guan C., Li Y. (2021). Effects of single and multiple transplantations of human umbilical cord mesenchymal stem cells on the recovery of ovarian function in the treatment of premature ovarian failure in mice. *Journal of Ovarian Research*.

[19] Besikcioglu H. E., Sarıbas G. S., Ozogul C. (2019). Determination of the effects of bone marrow derived mesenchymal stem cells and ovarian stromal stem cells on follicular maturation in cyclophosphamide induced ovarian failure in rats, Taiwan. *Journal of Obstetrics and Gynaecology*.

[20] Badawy A., Sobh M. A., Ahdy M., Abdelhafez M. S. (2017). Bone marrow mesenchymal stem cell repair of cyclophosphamide-induced ovarian insufficiency in a mouse model. *International Journal of Women’s Health*.

[21] Kilic S., Pinarli F., Ozogul C., Tasdemir N., Naz Sarac G., Delibasi T. (2014). Protection from cyclophosphamide-induced ovarian damage with bone marrow-derived mesenchymal stem cells during puberty. *Gynecological Endocrinology*.

[22] Khanmohammadi N., Sameni H. R., Mohammadi M. (2018). Effect of transplantation of bone marrow stromal cell-conditioned medium on ovarian function, morphology and cell death in cyclophosphamide-treated rats. *Cell Journal*.

[23] Lai D., Wang F., Yao X., Zhang Q., Wu X., Xiang C. (2015). Human endometrial mesenchymal stem cells restore ovarian function through improving the renewal of germline stem cells in a mouse model of premature ovarian failure. *Journal of Translational Medicine*.

[24] Feng P., Li P., Tan J. (2019). Human menstrual blood-derived stromal cells promote recovery of premature ovarian insufficiency via regulating the ECM-dependent FAK/AKT signaling. *Stem Cell Reviews and Reports*.

[25] Yoon S. Y., Yoon J. A., Park M. (2020). Recovery of ovarian function by human embryonic stem cell-derived mesenchymal stem cells in cisplatin-induced premature ovarian failure in mice. *Stem Cell Research & Therapy*.

[26] Bahrehbar K., Valojerdi M. R., Esfandiari F., Fathi R., Hassani S. N., Baharvand H. (2020). Human embryonic stem cell-derived mesenchymal stem cells improved premature ovarian failure. *World Journal of Stem Cells*.

[27] Liu R., Zhang X., Fan Z. (2019). Human amniotic mesenchymal stem cells improve the follicular microenvironment to recover ovarian function in premature ovarian failure mice. *Stem Cell Research & Therapy*.

[28] Huang B., Qian C., Ding C., Meng Q., Zou Q., Li H. (2019). Fetal liver mesenchymal stem cells restore ovarian function in premature ovarian insufficiency by targeting MT1. *Stem Cell Research & Therapy*.

[29] Sun M., Wang S., Li Y. (2013). Adipose-derived stem cells improved mouse ovary function after chemotherapy-induced ovary failure. *Stem Cell Research & Therapy*.

[30] Çil N., Mete G. A. (2021). The effect of adipose-derived mesenchymal stem cell treatment on mTOR and p-mTOR expression in ovarian damage due to cyclophosphomide. *Reproductive Toxicology*.

[31] Li J., Yu Q., Huang H. (2018). Human chorionic plate-derived mesenchymal stem cells transplantation restores ovarian function in a chemotherapy-induced mouse model of premature ovarian failure. *Stem Cell Research & Therapy*.

[32] Eslami N., Bahrebar K., Esfandiari F. (2023). Regenerative potential of different extracellular vesicle subpopulations derived from clonal mesenchymal stem cells in a mouse model of chemotherapy-induced premature ovarian failure. *Life Sciences*.

[33] Shin E. Y., Kim D. S., Lee M. J. (2021). Prevention of chemotherapy-induced premature ovarian insufficiency in mice by scaffold-based local delivery of human embryonic stem cell-derived mesenchymal progenitor cells. *Stem Cell Research & Therapy*.

[34] Yan L., Wu Y., Li L. (2020). Clinical analysis of human umbilical cord mesenchymal stem cell allotransplantation in patients with premature ovarian insufficiency. *Cell Proliferation*.

[35] Yang Y., Lei L., Wang S. (2019). Transplantation of umbilical cord–derived mesenchymal stem cells on a collagen scaffold improves ovarian function in a premature ovarian failure model of mice. *In Vitro Cellular & Developmental Biology Animal*.

[36] Ding L., Yan G., Wang B. (2018). Transplantation of UC-MSCs on collagen scaffold activates follicles in dormant ovaries of POF patients with long history of infertility. *Science China Life Sciences*.

[37] Fu X., He Y., Xie C., Liu W. (2008). Bone marrow mesenchymal stem cell transplantation improves ovarian function and structure in rats with chemotherapy-induced ovarian damage. *Cytotherapy*.

[38] Golchin A., Chatziparasidou A., Ranjbarvan P., Niknam Z., Ardeshirylajimi A. (2021). Embryonic stem cells in clinical trials: current overview of developments and challenges. *Advances in Experimental Medicine & Biology*.

[39] Igboeli P., El Andaloussi A., Sheikh U. (2020). Intraovarian injection of autologous human mesenchymal stem cells increases estrogen production and reduces menopausal symptoms in women with premature ovarian failure: two case reports and a review of the literature. *Journal of Medical Case Reports*.

[40] Wang L., Huang S., Li S. (2019). Efficacy and safety of umbilical cord mesenchymal stem cell therapy for rheumatoid arthritis patients: a prospective phase I/II study. *Drug Design, Development and Therapy*.

[41] Hwang J. J., Rim Y. A., Nam Y., Ju J. H. (2021). Recent developments in clinical applications of mesenchymal stem cells in the treatment of rheumatoid arthritis and osteoarthritis. *Frontiers in Immunology*.

[42] Nagamatsu G., Shimamoto S., Hamazaki N., Nishimura Y., Hayashi K. (2019). Mechanical stress accompanied with nuclear rotation is involved in the dormant state of mouse oocytes. *Science Advances*.

[43] Kreeger P. K., Deck J. W., Woodruff T. K., Shea L. D. (2006). The in vitro regulation of ovarian follicle development using alginate-extracellular matrix gels. *Biomaterials*.

[44] Mansouri V., Salehi M., davood Omrani M., Niknam Z., Ardeshirylajimi A. (2017). Collagen-alginate microspheres as a 3D culture system for mouse embryonic stem cells differentiation to primordial germ cells. *Biologicals*.

[45] Lai J. J., Chau Z. L., Chen S. Y. (2022). Exosome processing and characterization approaches for research and technology development. *Advanced Science*.

[46] Andronico F., Battaglia R., Ragusa M., Barbagallo D., Purrello M., Di Pietro C. (2019). Extracellular vesicles in human oogenesis and implantation. *International Journal of Molecular Sciences*.

[47] Diez-Fraile A., Lammens T., Tilleman K. (2014). Age-associated differential microRNA levels in human follicular fluid reveal pathways potentially determining fertility and success of in vitro fertilization. *Human Fertility*.

[48] Thabet E., Yusuf A., Abdelmonsif D. A., Nabil I., Mourad G., Mehanna R. A. (2020). Extracellular vesicles miRNA-21: a potential therapeutic tool in premature ovarian dysfunction. *Molecular Human Reproduction*.

[49] Liu M., Qiu Y., Xue Z. (2020). Small extracellular vesicles derived from embryonic stem cells restore ovarian function of premature ovarian failure through PI3K/AKT signaling pathway. *Stem Cell Research & Therapy*.

[50] Xiao G. Y., Cheng C. C., Chiang Y. S., Cheng W. T. K., Liu I. H., Wu S. C. (2016). Exosomal miR-10a derived from amniotic fluid stem cells preserves ovarian follicles after chemotherapy. *Scientific Reports*.

[51] Nazdikbin Yamchi N., Ahmadian S., Mobarak H. (2023). Amniotic fluid-derived exosomes attenuated fibrotic changes in POI rats through modulation of the TGF-*β*/Smads signaling pathway. *Journal of Ovarian Research*.

[52] Geng B., Chen H., Zou G. (2022). Human amniotic fluid mesenchymal stem cell-derived exosomes inhibit apoptosis in ovarian granulosa cell via miR-369-3p/YAF2/PDCD5/p53 pathway. *Oxidative Medicine and Cellular Longevity*.

[53] Ding C., Zhu L., Shen H. (2020). Exosomal miRNA-17-5p derived from human umbilical cord mesenchymal stem cells improves ovarian function in premature ovarian insufficiency by regulating SIRT7. *Stem Cells*.

[54] Tian G., Yi C., Min H., Ying D. (2022). Human umbilical cord mesenchymal stem cell-derived extracellular vesicles carrying MicroRNA-29a improves ovarian function of mice with primary ovarian insufficiency by targeting HMG-box transcription factor/wnt/*β*- catenin signaling. *Disease Markers*.

[55] Qu Y., Liu L., Cui Y. (2022). miR-126-3p containing exosomes derived from human umbilical cord mesenchymal stem cells promote angiogenesis and attenuate ovarian granulosa cell apoptosis in a preclinical rat model of premature ovarian failure. *Stem Cell Research & Therapy*.

[56] Yang Z., Du X., Wang C. (2019). Therapeutic effects of human umbilical cord mesenchymal stem cell-derived microvesicles on premature ovarian insufficiency in mice. *Stem Cell Research & Therapy*.

[57] Pu A., Zhang L., Zhang P. (2023). Human UC-MSC-derived exosomes facilitate ovarian renovation in rats with chemotherapy-induced premature ovarian insufficiency. *Frontiers in Endocrinology*.

[58] Huang B., Lu J., Ding C., Zou Q., Wang W., Li H. (2018). Exosomes derived from human adipose mesenchymal stem cells improve ovary function of premature ovarian insufficiency by targeting SMAD. *Stem Cell Research & Therapy*.

[59] Yang M., Lin L., Sha C. (2020). Bone marrow mesenchymal stem cell-derived exosomal miR-144-5p improves rat ovarian function after chemotherapy-induced ovarian failure by targeting PTEN. *Laboratory Investigation*.

[60] Sun B., Ma Y., Wang F., Hu L., Sun Y. (2019). MIR-644-5p carried by bone mesenchymal stem cell-derived exosomes targets regulation of p53 to inhibit ovarian granulosa cell apoptosis. *Stem Cell Research & Therapy*.

[61] Zhang S., Huang B., Su P. (2021). Concentrated exosomes from menstrual blood-derived stromal cells improves ovarian activity in a rat model of premature ovarian insufficiency. *Stem Cell Research & Therapy*.

[62] Song J. T., Aixin, Zhang S. (2023). Exosomes derived from menstrual blood stromal cells ameliorated premature ovarian insufficiency and granulosa cell apoptosis by regulating SMAD3/AKT/MDM2/P53 pathway via delivery of thrombospondin-1, Biomed. *Pharma*.

[63] Cha J. M., Shin E. K., Sung J. H. (2018). Efficient scalable production of therapeutic microvesicles derived from human mesenchymal stem cells. *Scientific Reports*.

[64] Navakanitworakul R., Hung W. T., Gunewardena S., Davis J. S., Chotigeat W., Christenson L. K. (2016). Characterization and small RNA content of extracellular vesicles in follicular fluid of developing bovine antral follicles. *Scientific Reports*.

[65] Li X., Xie J., Wang Q., Cai H., Xie C., Fu X. (2020). MiR-21 and pellino-1 expression profiling in autoimmune premature ovarian insufficiency. *Journal of Immunology Research*.

[66] Zhang Q., Sun J., Huang Y. (2019). Human amniotic epithelial cell-derived exosomes restore ovarian function by transferring MicroRNAs against apoptosis. *Molecular Therapy- Nucleic Acids*.

[67] Niu S., Yu F., Luo X., Chen (2022). Human umbilical cord mesenchymal stem cells improve premature ovarian failure through cell apoptosis of miR-100-5p/NOX4/NLRP3. *BioMed Research International*.

[68] Li Z., Zhang M., Zheng J. (2021). Human umbilical cord mesenchymal stem cell-derived exosomes improve ovarian function and proliferation of premature ovarian insufficiency by regulating the Hippo signaling pathway. *Frontiers in Endocrinology*.

[69] soo Park H., Chugh R. M., El Andaloussi A. (2021). Human BM-MSC secretome enhances human granulosa cell proliferation and steroidogenesis and restores ovarian function in primary ovarian insufficiency mouse model. *Scientific Reports*.

[70] soo Park H., Chugh R. M., Seok J. (2023). Comparison of the therapeutic effects between stem cells and exosomes in primary ovarian insufficiency: as promising as cells but different persistency and dosage. *Stem Cell Research & Therapy*.

[71] Riau A. K., Ong H. S., Yam G. H. F., Mehta J. S. (2019). Sustained delivery system for stem cell-derived exosomes. *Frontiers in Pharmacology*.

